# Attenuation of hypertension by C-fiber stimulation of the human median nerve and the concept-based novel device

**DOI:** 10.1038/s41598-018-33402-1

**Published:** 2018-10-08

**Authors:** Se Kyun Bang, Yeonhee Ryu, Suchan Chang, Chae Kwang Im, Jong Han Bae, Young Seob Gwak, Chae Ha Yang, Hee Young Kim

**Affiliations:** 1 College of Korean Medicine, Daegu Haany University, Daegu, 42158 South Korea; 2 Clinical Medicine Division, Korea Institute of Oriental Medicine, Daejeon, 34054 South Korea; 3 Department of Physics, Yeungnam University, Gyeongsan, Gyeongbukdo, 38541 South Korea

## Abstract

High blood pressure (BP) is a highly controllable risk factor for cardiovascular diseases; however, awareness of this condition and the rates of controlled hypertension are low. Experimental animal studies have shown that stimulation of the median nerve or PC6 acupoint over the wrist has effects on cardiovascular activities, including reductions in systolic and diastolic BPs. A proof-of-concept study was conducted in humans to investigate whether stimulation of median nerve near PC6 acupoint decreased high BP, identify the optimal stimulation parameters for the BP-lowering effects of median nerve stimulation, and determine the specific peripheral nerves or types of afferent fibers mediating the BP-lowering effects. Median nerve stimulation was carried out bilaterally or unilaterally with different stimulation parameters, and the BP and heart rate were monitored. The afferent mechanisms underlying the effects of median nerve stimulation on hypertension were investigated via microneurography, A-fiber blocking experiments, and localized chemical or electrical stimulation. Bilateral median nerve stimulation at either low or high frequencies produced profound but transient reductions in systolic BP, which were elicited when median nerve stimulation was unilaterally applied at interelectrode distances of 2 and 4 cm. Systolic BP was also reduced by electrical stimulation of the thumb on the palm side. Although microneurographic recordings revealed the excitation of both A- and C-fibers following median nerve stimulation, the median nerve-mediated reductions in BP were not affected by A-fiber blockade, and they were mimicked by the activation of C-fibers with capsaicin. The present results indicate that activation of C-fibers in the median nerve generates BP-lowering effects in humans. Based on our clinical study, an optimized median nerve stimulator was built and combined with a wrist BP monitor for simultaneous BP measurements and median nerve stimulation.

## Introduction

Hypertension is one of the most common and most serious diseases and is estimated to impact more than 1 billion people worldwide^1^. High blood pressure (BP) is a significant risk factor because it often leads to fatal outcomes, such as heart attack, renal failure and stroke^2^. The mainstay of the medical management of hypertension includes a number of commonly used antihypertensive drugs. However, certain hypertensive patients appear to be resistant to multiple antihypertensive drugs, and antihypertensive drugs frequently cause side effects, such as fatigue, dizziness and reduced exercise tolerance^3^. Approximately 50% of newly diagnosed hypertensive patients discontinue their antihypertensive medications 6 months after the initial prescription, and up to 25% of patients with hypertension stop taking their antihypertensive drugs^4,5^.

While antihypertensive drugs are commonly prescribed for hypertension, there has been interest in device-based therapies designed to nonpharmacologically modulate cardiovascular functions. Therapeutic interventions, including deep brain stimulation (DBS) of the periaqueductal gray matter (PAG), vagus nerve stimulation, baroreflex activation therapy, renal denervation or carotid body ablation, are known to lower or raise BP and/or heart rate (HR)^6^. However, those methods require invasive procedures or are not amenable to long-term ambulatory use outside the laboratory or the hospital during normal daily activities. In contrast, external nerve stimulation has the advantages of noninvasiveness, ease of use and minimal side effects after long-term use^7,8^. Experimental median nerve stimulation (i.e., electroacupuncture at PC6 acupoint) in animals relieves cardiovascular disorders, such as hypertension, myocardial ischemia, myocardial infarction, and arrhythmia, by inducing the release of endogenous opioids and modulating the autonomic nervous system^9,10^. Although animal studies have suggested that external median nerve stimulation can suppress elevated BP, the effects of this stimulation and the afferent mechanisms in human subjects are unclear (see the review^6^).

The aims of this study on human subjects are to (1) determine whether median nerve stimulation alleviates hypertension, (2) determine which stimulation conditions are most effective at reducing BP, and (3) investigate the nerve and the types of afferent fibers mediating the antihypertensive effects. Based on the results from the aforementioned proof-of-concept study, the present study finally aimed to develop a noninvasive, wrist-worn neuromodulation device for lowering BP.

## Results

### Experiment 1: Effects of varying frequencies of transcutaneous median nerve stimulation (TMNS) on hypertension

**Figure 1:**
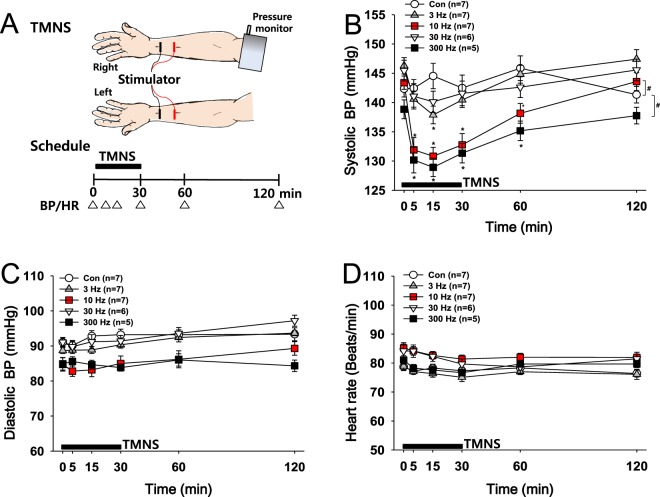
Effects of TMNS applied at different frequencies on hypertension. (**A**) Experimental scheme. Transcutaneous median nerve stimulation (TMNS) and measurements of blood pressure (BP) and heart rate (HR). (**B**–**D**) Changes in systolic BP (**B**), diastolic BP (**C**) and HR (**D**) following the application of bilateral TMNS at frequencies of 3, 10, 30 or 300 Hz. The application of TMNS at 10 or 300 Hz transiently reversed the changes in systolic BP compared to the control (Con), although neither diastolic BP nor HR were altered. *p < 0.05 vs. the values recorded before TMNS (Time 0); and ^#^p < 0.05 vs. the Con group.

TMNS was applied bilaterally for 30 min at frequencies of 3, 10, 30 or 300 Hz to study the effects of TMNS on hypertension and determine the optimal stimulation frequency (Fig. 1A). While the control group (Con; no TMNS) showed high systolic BP (over 140 mmHg), the administration of TMNS at frequencies of 3, 10 and 300 Hz significantly reduced the systolic BP compared to the basal systolic BP, with the greatest effect observed at 15 min after the initiation of stimulation. The significant reduction in the systolic BP persisted for up to 60 min, and the BP returned to basal levels 120 min after TMNS. The application of TMNS at either 10 or 300 Hz exerted a profound but transient effect on reducing the systolic BP compared to the effects of other treatments (Con vs. 3 or 30 Hz; two-way repeated measures ANOVA: group factor, F = 35.235, p < 0.001; time factor, F = 18.021, p < 0.001; Fig. 1B). In contrast, no significant differences in diastolic BP or HR were observed between groups before and after TMNS (Fig. 1C,D). Although the reductions in systolic BP in the 10 and 300 Hz groups were similar, the volunteers reported that 10-Hz TMNS was more comfortable than 300-Hz TMNS. Accordingly, TMNS was applied at 10 Hz in the subsequent experiments.

### Experiment 2: Comparison of the effects of unilateral or bilateral TMNS on hypertension

**Figure 2:**
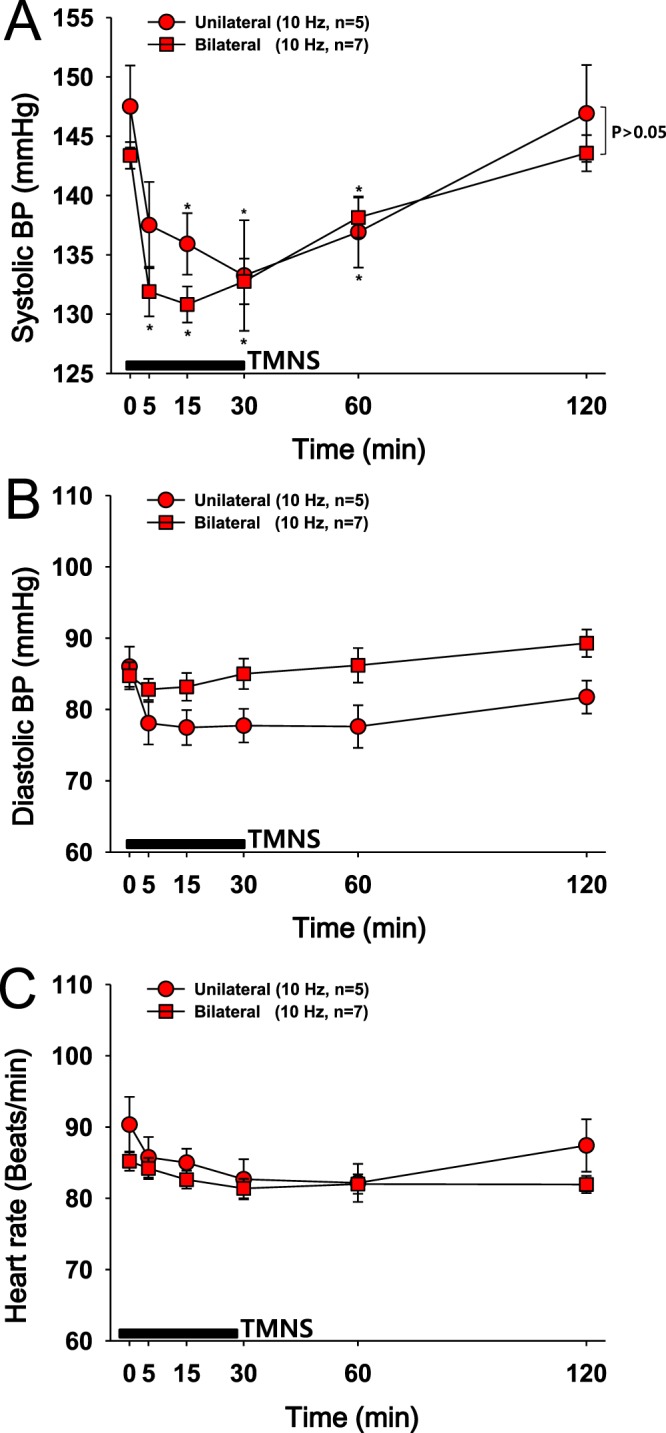
Comparison of the effects of unilateral or bilateral TMNS on hypertension. (**A**–**C**) Changes in systolic blood pressure (BP) (**A**), diastolic BP (**B**) and HR (**C**) following the application of unilateral or bilateral TMNS. The data for bilateral TMNS shown in (**A**–**C**) are duplicated from the 10-Hz group shown in Fig. 1 according to recommendations of the IRB. The application of unilateral (left hand) or bilateral TMNS at a frequency of 10 Hz significantly decreased systolic BP compared to the values recorded before TMNS. A significant difference was not observed between the two groups (unilateral vs. bilateral). *p < 0.05 vs. the values obtained before TMNS (Time 0).

The effects of unilateral TMNS on systolic BP were investigated by applying TMNS to the left hand for 30 min at a frequency of 10 Hz, and the effects were compared with the effects of bilateral TMNS (10 Hz in experiment 1). Compared to the baseline, unilateral TMNS significantly reduced the systolic BP from 147.5 ± 3.45 to 133.25 ± 4.6 mmHg, with the greatest effect observed at 30 min after stimulation (one-way repeated ANOVA: F = 11.129, p < 0.001). Bilateral TMNS decreased the systolic BP from 143.38 ± 1.12 to 130.8 ± 1.52 mmHg, with the greatest effect observed at 15 min after stimulation. A significant difference in the reduction of systolic BP was not observed between the two groups (unilateral and bilateral TMNS) (Fig. 2A). In addition, no or only a minimal decrease in diastolic BP (Fig. 2B) and HR (Fig. 2C) was observed after unilateral or bilateral TMNS. Because unilateral TMNS itself generated antihypertensive effects as effectively as bilateral TMNS, TMNS was applied to only the left hand in the subsequent experiments.

### Experiment 3: Effects of the interelectrode distance for TMNS on hypertension

**Figure 3:**
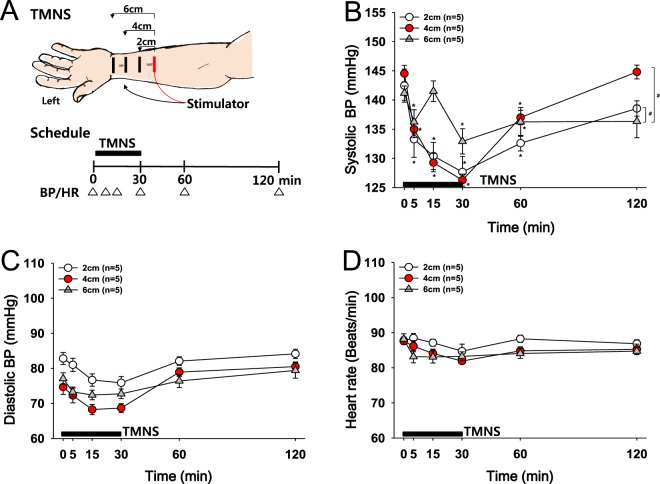
Effects of the interelectrode distance for TMNS on hypertension. (**A**) Experimental scheme. Placement of electrodes (black, anode; red, cathode) and measurements of blood pressure (BP) and heart rate (HR). (**B**–**D**) Changes in systolic BP (**B**), diastolic BP (**C**) and HR (**D**) following the application of unilateral TMNS (10 Hz, left hand) at distances of 2, 4 or 6 cm between the two electrodes. *p < 0.05 vs. the values obtained before TMNS (Time 0); ^#^p < 0.05 vs. the 6 cm-group.

The optimal distance of two electrodes for TMNS was determined by comparing the effects of the distance from the active electrode (red, +) to the reference electrode (black, −) on systolic BP. TMNS was applied to the left forehand at a frequency of 10 Hz (Fig. 3A). A significant and consistent reduction in systolic BP was produced by TMNS applied at a distance of 2 cm (142.46 ± 2.83 to 127.66 mmHg) and 4 cm (144.53 ± 1.42 to 126.26 ± 1.03 mmHg) compared to TMNS applied at 6 cm (Fig. 3B; two-way repeated measures ANOVA: group factor, F = 35.3, p < 0.001; time factor, F = 17.810, p < 0.001). No or only slight changes in diastolic BP or HR were observed after unilateral TMNS. The application of TMNS with electrodes placed 2 and 4 cm apart produced similar patterns of significant reductions in systolic BP. However, an interelectrode distance of 4 cm was easier to use, as it allowed for a more stable placement and easier maintenance of electrodes during stimulation than a distance of 2 cm. Thus, an interelectrode distance of 4 cm on the left forehand and TMNS at 10 Hz were used in the subsequent experiments.

### Experiment 4: Effects of median nerve stimulation on reducing BP

**Figure 4:**
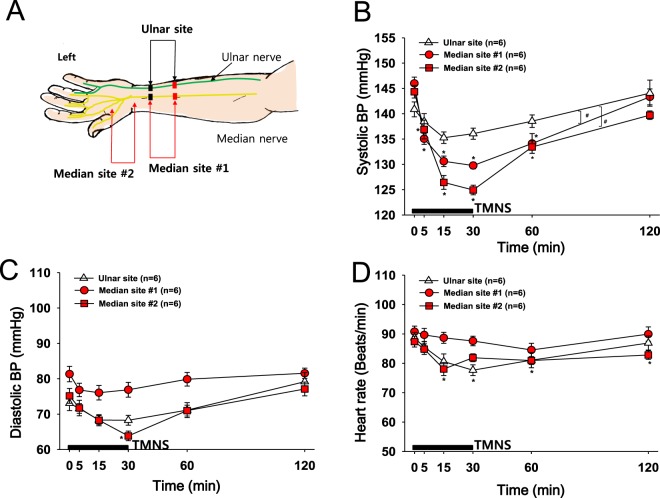
Effects of electrical stimulation of the skin over the median or ulnar nerve on hypertension. (**A**) Experimental scheme. Location of electrodes for stimulation of the median nerve (Median site #1 and #2) or ulnar nerve (Ulnar site) sites and measurement of BP and HR. (**B**–**D**) Changes in systolic BP (**B**), diastolic BP (**C**) and HR (**D**) following electrical stimulation (10 Hz) of the skin innervating the median or ulnar nerve on the left hand. Decreases in systolic BP were more profound following the stimulation of the median nerve sites (Median site #1 & #2) than the stimulation of the ulnar nerve site (Ulnar site; A). Electrical stimulation of the palm side of the thumb (Median site #2) also tended to decrease diastolic BP and HR. *p < 0.05 vs. the value obtained before TMNS (Time 0). ^**#**^p < 0.05 vs. the Ulnar site.

As horizontal stimulation with long strip electrodes (1 cm × 5 cm) attached to the skin of the medial aspect of the forehand might activate the median nerve together with the ulnar nerve and thus produce antihypertensive effects, smaller electrodes (1.5 cm × 1.5 cm) were used to selectively stimulate either the median nerve or ulnar nerve. Electrodes were attached to the left hand as shown in Fig. 4A, and the subjects were electrically stimulated at a frequency of 10 Hz. Although ulnar nerve stimulation slightly decreased the systolic BP, a more profound decrease in systolic BP was observed with median nerve stimulation (Median site #1) than with ulnar nerve stimulation (Ulnar site, p = 0.032). The effect of median nerve stimulation on reducing hypertension was further confirmed by our experiment showing that electrical stimulation of the skin on the palm side of the thumb (median nerve territory; Median site #2) mimicked the effects of TMNS resulting from stimulation at Median site #1 (Fig. 4B). Slight but significant decreases in diastolic BP and HR compared with the baseline values were observed when electrical stimulation was applied to the skin of the palm side of the thumb.

### Experiment 5: Fiber types activated during TMNS

**Figure 5:**
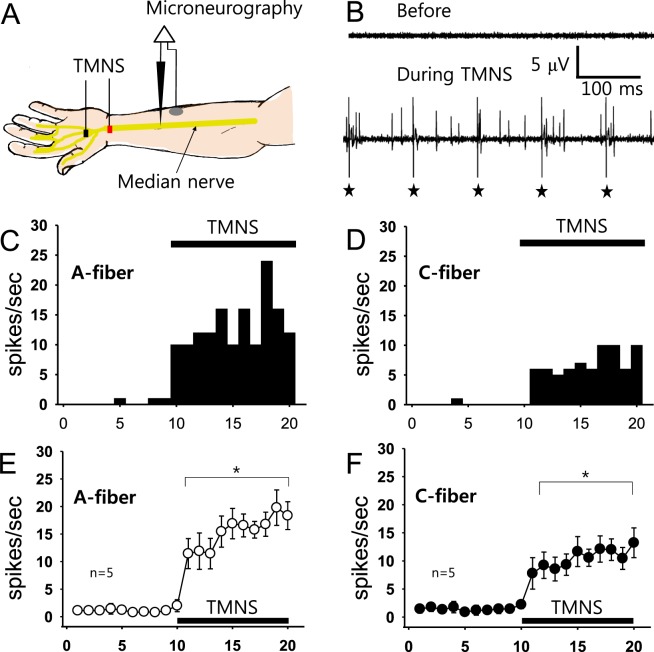
Microneurographic recordings of the median nerve during TMNS. (**A**) Experimental scheme for median nerve microneurography and TMNS (10 Hz, left hand). (**B**) Representative trace of fiber discharges during TMNS. Asterisks indicate electrical artifacts from 10-Hz TMNS. (**C**–**E**) A representative histogram (**C**) and mean values (**E**, n = 5 persons) of A-fiber discharges before and after TMNS. (**D** and **F**) Representative histogram (**D** and **F**) mean values (**F**, n = 5 persons) of C-fiber discharges before and after TMNS. *p < 0.05 vs. mean values obtained before TMNS (Times 1–10).

Microneurography of the median nerve was performed to identify the type of nerve fibers activated during TMNS (n = 5, Fig. 5A). When a single-unit discharge from A- or C-fibers was isolated (Fig. 5B), TMNS was applied for 10 seconds at a frequency of 10 Hz to the left hand. Figure 5C,D shows a representative example indicating that TMNS simultaneously activated A-fibers (Fig. 5C) and C-fibers (Fig. 5D). When TMNS was applied for 10 seconds as shown in Fig. 5A, the single-unit discharges of both the A- and C-fibers were significantly evoked (Fig. 5E,F) compared to the values obtained before treatment, indicating that both the A- and C-fibers of the median nerve were activated by TMNS.

### Experiment 6: Effect of an AFB (A-fiber block) on reducing BP during TMNS

#### Experiment 6-1: Induction and monitoring of an AFB of the median nerve

**Figure 6:**
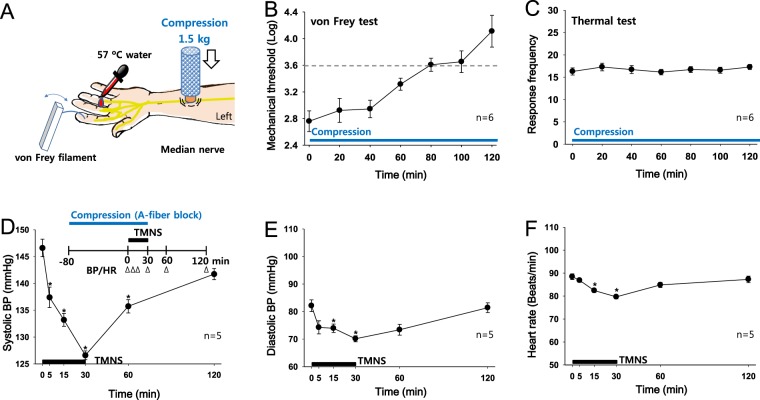
Effects of a median nerve A-fiber block (AFB) on TMNS-induced antihypertensive effects. (**A**–**C**) AFB of the median nerve induced by compression in 6 subjects. Compression over the median nerve was performed with a 1.5-kg weight (**A**), and mechanical (**B**) and thermal sensitivities (**C**) were evaluated using von Frey filaments and warm water (57 °C), respectively. Mechanical thresholds were increased slowly, reaching a log value of 3.61 at 80 min after compression, whereas the thermal sensitivity in response to the 57 °C water was not changed after compression for up to 120 min. (**D**–**F)** Effect of the median nerve AFB on TMNS-induced antihypertensive effects on hypertensive subjects (n = 5). Changes in systolic BP (**D**), diastolic BP (**E**) and HR (**F**) following the application of TMNS (unilateral, 10 Hz) to the left hand subjected to 80 min of compression to establish a median nerve AFB. *p < 0.05 vs. the values obtained before TMNS.

A custom-made compressor with a 1.5-kg weight was placed over the left hand of subjects (n = 6, Fig. 6A) between the ligaments of the flexor carpi radialis and the palmaris longus and 7 cm proximal to the wrist crease to determine the time required to establish an AFB of the median nerve by compression. Mechanical (A-fiber) and thermal (C-fiber) sensitivities were evaluated every 20 min using von Frey filaments and warm water (57 °C), respectively. Since a logarithmic bending force of 3.61 (0.4-gram force) is usually used to verify an AFB in humans, A-fiber conduction was considered significantly affected at a value greater than 3.61^11^. Mechanical sensitivity slowly increased over 120 min after compression and reached a logarithmic value of 3.61 at 80 min (Fig. 6B). However, thermal sensitivity was not altered over the 120-min period (Fig. 6C), indicating that C-fiber conduction was intact. Thus, a time point of 80 min after the initiation of compression was used to block median nerve A-fibers in the next experiment.

#### Experiment 6-2: Effect of an AFB on reducing elevated BP during TMNS

Five hypertensive subjects were administered an AFB for 80 min followed by 30 min of TMNS to the skin of the palm side of the thumb to explore whether A-fibers mediated the effects of TMNS on hypertension, and the effects were compared to the values obtained before TMNS. TMNS significantly decreased the systolic BP (148.60 ± 1.63 to 126.60 ± 0.82 mmHg; Fig. 6D), with the greatest effect observed at 30 min after TMNS (Friedman repeated measures analysis of variance on rank: chi-square = 62.871 with 5 degrees of freedom, p < 0.001). The antihypertensive effect of TMNS was not blocked by the compression that inhibited A-fiber conduction. Thus, transient reversals of hypertension by TMNS may be mediated by the activation of C-fibers and not A-fibers.

### Experiment 7: Effect of a topical capsaicin treatment on BP and HR in hypertensive subjects

**Figure 7:**
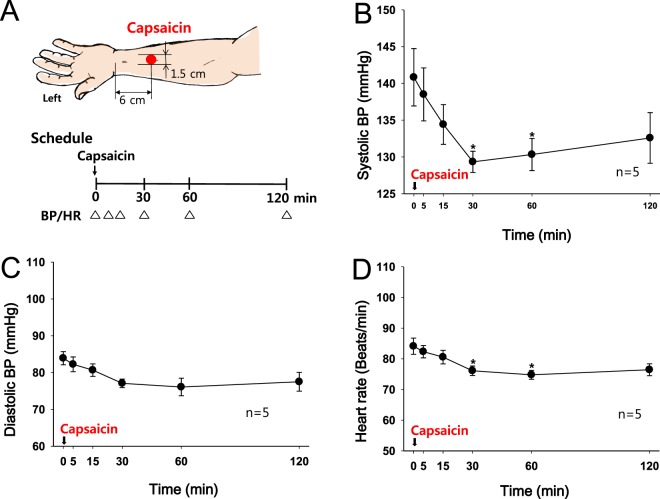
Effect of a topical capsaicin treatment to the skin over the median nerve on hypertension. (**A**) Experimental scheme. (**B**–**D**) Changes in systolic BP (**B**), diastolic BP (**C**) and HR (**D**) following the topical capsaicin treatment over the median nerve of the left hand. *p < 0.05 vs. the values obtained before the capsaicin treatment.

Capsaicin (0.1% in ethanol) was applied to the skin over the median nerve 6 cm proximal to the wrist crease to confirm that C-fibers mediated the reduction in systolic BP induced by median nerve stimulation (Fig. 7A). The systolic BP slowly decreased over 30 min, and the effect persisted for up to 120 min (Fig. 7B, one-way repeated measures ANOVA F = 2.452, p = 0.035) compared to basal BP. Diastolic BP (Fig. 7C) and HR (Fig. 7D) were slightly decreased following the capsaicin treatment.

### Experiment 8: Prototype device consisting of a TMNS unit and a wrist BP monitor

**Figure 8:**
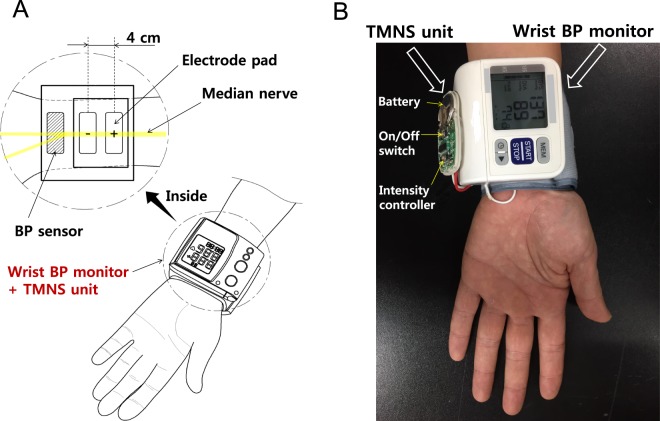
Prototype wrist-worn BP neuromodulation device. (**A**) Schematic of a TMNS-coupled BP monitor. (**B**) Prototype device constructed by attaching the newly developed TMNS unit to a commercial wrist BP monitor. TMNS, transcutaneous median nerve stimulation; and BP, blood pressure.

We examined whether repetitive TMNS could improve cardiac functions in hypertensive subjects. In the 6 participants who received repetitive TMNS by completing all test sessions over approximately 5 months, basal systolic BP, diastolic BP and HR were analyzed. Although the basal values showed fluctuations, the systolic BP values dropped significantly from approximately 155 mmHg to approximately 140 mmHg at 4 weeks, and this significant decrease was maintained for up to 14 weeks (one-way repeated measures ANOVA, F = 6.506, p < 0.001). A significant reduction in diastolic BP was also observed 8 weeks after repetitive TMNS compared to the initial diastolic BP values (one-way repeated measures ANOVA, F = 5.801, p < 0.001). In contrast, HR was not altered over the 14 weeks (Supplementary Figure 1), suggesting the reduction of elevated BP by repetitive TMNS in hypertensive subjects. Based on the results of the aforementioned experiments, we developed a small TMNS unit with the following parameters: a frequency of 10 Hz, triangular pulses of 0.2 ms in duration and a 30-minute treatment per session. The unit was attached to the main body of a portable wrist BP monitor. Two electrode pads (1-cm wide and 5-cm long) connecting the TMNS unit were fixed at an interelectrode distance of 4 cm on the internal surface of the BP monitor wrist cuff. The electrode pads were positioned to be located on the skin over the median nerve when the wrist cuff was wrapped for BP measurements (Fig. 8A). This device enabled simultaneous BP measurements and median nerve stimulation (Fig. 8B).

## Discussion

Electrical stimulation applied to the skin over the median nerve (denoted as TMNS) significantly lowered the systolic BP. Unilateral TMNS elicited effects similar to bilateral stimulation. The transient reversals of hypertension by left TMNS were generated with an interelectrode distance of 2 or 4 cm. The effects of stimulation of the median nerve on reducing hypertension were confirmed by the data showing that electrical stimulation of the skin over the palm side of the thumb or over the median nerve exerted the same effect. Microneurography revealed the activation of both A- and C-fibers of the median nerve during TMNS. The effects of TMNS on hypertension were not affected by the selective blockade of A-fibers but were reproduced by the application of capsaicin, a C-fiber activator, to the skin over the median nerve. Thus, TMNS reduced systolic BP by activating C-fibers in the median nerve. Based on the results from the current proof-of-concept study, a TMNS unit for lowering BP was built and combined with a wrist BP monitor.

### Either low- or high-frequency electrical nerve stimulation produces a transient reduction in BP in humans

Afferent nerve stimulation, such as external electric nerve stimulation, has been widely used to treat pain. These types of electrical stimulations produce a variety of cardiovascular responses, including a reduction in systolic or diastolic BP, changes in HR and modulation of parasympathetic and sympathetic responses, depending on the stimulation parameters, such as the frequency, intensity, pulse duration and positioning of the electrodes^7,12–14^. The stimulation frequency is broadly classified as high (>50 Hz) or low (≤10 Hz) frequency^12^, and the effects of high- and low-frequency stimulation on cardiovascular responses have been investigated in human subjects. Although some controversy exists concerning the effect of nerve stimulation on BP, low-frequency peripheral nerve stimulation has been reported to produce systolic and diastolic BP changes in hypertensive patients^15,16^. High-frequency stimulation also decreases the HR and systolic arterial pressure in healthy subjects^17^ and attenuates exercise-induced BP and vasoconstriction in healthy subjects^18^. These results are similar to our present findings that electrical nerve stimulation at either low (10 Hz) or high (300 Hz) frequencies produced a transient reduction in systolic BP in hypertensive subjects. Either high- or low-frequency peripheral nerve stimulation increases the release of endogenous opioids, such as β-endorphins, in the cerebrospinal fluid and bloodstream in humans^19,20^. These opioid peptides are critically involved in the pathogenesis of hypertension^21^, and the systemic administration of endorphins induces a transient reduction in arterial BP in rats^22^. Thus, in the present study, the transient reduction in BP induced by low- or high-frequency stimulation of the median nerve in hypertensive subjects might have been mediated by the increased release of endogenous opioids.

### Median nerve stimulation alleviates hypertension in humans

According to experimental animal studies, stimulation of the median nerve inhibits sympatho-excitatory cardiovascular responses and thereby decreases cardiac sympathetic drive and BP^23,24^. When the median nerves were exposed in the forelimbs of a cat model of cardiac ischemia and directly stimulated with bipolar electrodes, bradykinin-induced elevations in systolic BP were attenuated, and the improved contractile function remained at a constant level for hours^24^. Similarly, electrical stimulation of acupoints, such as PC5 and PC6 located over the median nerve, reduces BP and decreases the incidence of ventricular tachycardia and fibrillation during reperfusion in rats^23^. While these previous animal studies have suggested that median nerve stimulation reverses hypertension^23,24^, the current study provides the first direct evidence of the afferent mechanism for the BP-lowering effects of median nerve stimulation on hypertensive human subjects. Electrical or chemical stimulation of C-fibers in the median nerve reduced the systolic BP. The reductions in systolic BP were more profound after localized stimulation of the median nerve than after stimulation of the ulnar nerve site, and the effects were reproduced by the electrical stimulation of the palm side of the thumb at the area overlying the branch of median nerve, suggesting that the median nerve mediated the TMNS-induced reduction of elevated BP in humans. Furthermore, the reduction in BP may exhibit stimulation-site specificity. In support of this hypothesis, the application of electroacupuncture at points overlying the median nerve was most effective in patients with reflex-induced hypertension in a previous study, while stimulation of points overlying the radial nerve or deep peroneal nerve was not effective or less effective^10^. Although the central mechanism by which median nerve stimulation reduces BP in humans remains to be elucidated, median nerve-mediated inhibition of BP may share similar neural pathways with the inhibitory effects of electroacupuncture applied to points near the median nerve on hypertension. The proposed pathways include the arcuate nucleus in the hypothalamus, ventral periaqueductal gray, and nucleus raphe obscurus, with a projection to the rostral ventrolateral medulla (rVLM), and they are thought to be modulated by the release of several neurotransmitters following stimulation. In these pathways, the release of endorphins, serotonin, and γ-aminobutyric acid following stimulation results in the inhibition of cardiac sympathetic neurons in the rVLM and alleviates hypertension^10^.

In the present study, median nerve stimulation significantly decreased systolic BP and tended to slightly decrease HRs. This finding may suggest a decrease in sympathetic neural outflow that mediates rapid and short-term changes in BP^25^. Previous studies have shown that somatic afferent stimulation such as median nerve stimulation or electroacupuncture causes the release of opioids and can inhibit elevated BP as well as sympathetic neural firing in the rVLM, an important nucleus in the regulation of sympathetic outflow^24,26–28^. The opioid peptides inhibit sympathetic outflow via the activation of μ-opioid receptors in the rVLM and decrease the sympathetic excitatory response induced by the activation of visceral afferents^24,26,27^. In addition, muscle sympathetic nerve activity (MSNA), which is composed of activity of the vasoconstrictor fibers innervating skeletal blood vessels, is known to play an important role in regulating BP. Activation of MSNA increases BP, while a reduction in MSNA decreases BP^29^. Since MSNA inhibition induces a decrease in BP, MSNA may also be attenuated during median nerve stimulation. However, median nerve stimulation caused a small decrease in diastolic BP. Therefore, we presumed that the significant decrease in systolic BP was likely associated with activation of μ-opioid receptors in the rVLM and a reduction in stroke volume, which must be verified.

### C-fibers mediate the TMNS-induced BP reduction in hypertensive human subjects

Transcutaneous nerve stimulation activates A-fibers, C-fibers, or both depending on the frequency and intensity^12^. According to our microneurographic recordings, both A- and C-fibers of the median nerve were activated when TMNS was applied at a high stimulation intensity (motor contraction) and low frequency (10 Hz), which is similar to the findings of a previous animal study^14^. Furthermore, an experimental median nerve AFB established prior to TMNS failed to prevent the BP-lowering effects of TMNS. In turn, the application of capsaicin, a C-fiber activator, to the skin over the median nerve produced TMNS-like effects on hypertension, suggesting a pivotal role of C-fibers in the reversal of hypertension induced by median nerve stimulation. Consistent with our findings, electroacupuncture (EA) at PC5-6 acupoints over the median nerve activates C- and Aδ-fibers in animal models to evoke cardiovascular effects^14^, and the cardiovascular effects of EA are diminished in rats depleted of C-fibers by neonatal treatment with capsaicin^30^. As shown in our recent study, cutaneous neurogenic inflammation caused by the activation of C-fibers during the development of hypertension is most frequently observed in the skin over the median nerve in rats^31^, which corresponds to the location on human skin where TMNS was applied in the present study. When areas of skin displaying neurogenic inflammation located over the median nerve are stimulated electrically at a high intensity and low frequency, the increase in BP is prevented in the rat model of immobilization-induced hypertension. Furthermore, the activation of C- and Aδ-fibers by injecting capsaicin (a transient receptor potential vanilloid 1 (TRPV1) agonist) or mustard oil (a TRP ankyrin 1 (TRPA1) agonist) into skin with neurogenic inflammation over the median nerve blocks the development of hypertension in rats^31^. Based on these findings, median nerve stimulation may require the activation of C-fibers to decrease systolic BP in hypertensive human subjects.

For the C-fiber stimulation of the median nerve in hypertensive patients, a TMNS unit for lowering BP was developed and coupled with a portable wrist BP monitor, which enabled simultaneous BP measurements and median nerve stimulation. However, more mechanistic studies, clinical trials and safety and side-effect evaluations must be performed before its clinical application. These antihypertensive effects were also achieved by electrical stimulation of several other nerves. Device-based stimulation of the renal sympathetic, vagus or carotid sinus nerves has been shown to reduce BP and has been proposed as an alternative treatment for hypertension. However, these nerves are highly heterogeneous in axonal composition for electrical stimulation, and the reports of side effects, such as complications from invasive surgical procedures, apnea, paresthesia, and voice alterations, limit the application of these procedures^6,32,33^. In our current study, electrical stimulation applied to the skin over the median nerve of the left hand effectively reduced systolic BP, and in clinical trials, adverse effects have not been reported; therefore, median nerve stimulation may be superior to the stimulation of other sites with respect to its noninvasiveness, convenience and long-term use (i.e., a wearable device).

In conclusion, our findings suggest that median nerve stimulation can produce reduction of elevated BP via activation of C-fibers in hypertensive human subjects. Based on the present results, a wrist-worn neuro-modulation device was developed. This device may assist hypertensive patients with lowering and monitoring their BP.

## Methods

### Subjects

The study was approved by the Institutional Review Board of Daegu Haany University (DHUMC-D-15010-PRO-03), registered at the Clinical Research Information Service of the Republic of Korea (CRIS; http://cris.nih.go.kr; KCT0001948; date of registration, 10/16/2015; date of first enrollment, 10/16/2015; study completion date, 02/29/2016) and performed according to the guidelines of the Declaration of Helsinki. The experimental procedures were explained to all participants, who provided informed consent. Twenty two volunteers were recruited and the 11 volunteers fulfilling the inclusion/exclusion criteria finally participated: 10 males and 1 female, mean age 33.56 ± 12.68 years, with grade 1 hypertension (average systolic pressure 140–159 and diastolic pressure 90–99 mmHg) but not taking anti-hypertensive medication (Supplementary Figure 2). The participants were asked to visit once per week for a total of 16 weeks, and they were randomly assigned to one of the treatment groups in each experiment (by a drawing from a masked card sequence arranged from a table of random numbers) except for the experiments shown in Figs 5 and 6A. All subjects were asked to refrain from food for 1 hour, alcohol for 12 hours and caffeine intake and smoking for 2 hours prior to the study. Testing was conducted at the same time of day (between 2:00 pm and 5:00 pm) in rooms set to 23 °C and 60% humidity.

### Measurements of BP and HR and transcutaneous electrical nerve stimulation

Upon arrival, the subjects were asked to rest for at least 30 min in a seated position prior to testing and advised not to cross their legs during the experiments. Systolic and diastolic BP and HR were measured from the right arm with a validated electronic sphygmomanometer (FT-500R, Jawon Medical, Kyungsan, South Korea) as described previously^34^. At each time point, the BP and HR were obtained from the average of two measurements. After measuring the resting BP and HR, the subjects received TMNS for 30 min. The BP and HR were measured at 5, 15, 30, 60 and 120 min after initiation of TMNS. For TMNS, electrode sites were precleaned with an alcohol swab to reduce skin resistance and electrically stimulated using a transcutaneous electrical nerve stimulator (a triangular pulse with a duration of 0.2 ms; OTS H-306, HAN-IL Co., South Korea). The stimulation intensity was adjusted to the maximum tolerable intensity that did not cause discomfort or pain.

## Experimental Protocols

### Experiment 1: Effects of varying frequencies of TMNS on hypertension

Two pairs of anodes and cathodes (1-cm wide and 5-cm long) were attached at 2 cm and 6 cm proximal to the transverse crease of the wrist, respectively that corresponds to location of PC6 acupoint in Oriental medicine. Electrical stimulation was applied bilaterally for 30 min at frequencies of 3, 10, 30 or 300 Hz. The control group (Con) underwent the same procedure as the TMNS group but without electrical stimulation. The stimulation time of 30 min was chosen based on a previous study^35^.

### Experiment 2: Comparison of the effects of unilateral or bilateral TMNS on hypertension

The location and size of electrodes were the same as in experiment 1 but TMNS was applied at a frequency of 10 Hz over the left forehand (unilateral). The effects of unilateral TMNS on BP and HR were compared with the effects of bilateral TMNS (10 Hz in experiment 1).

### Experiment 3: Effects of the interelectrode distance for TMNS on hypertension

The effects of the electrode position on BP and HR were evaluated by applying TMNS at 3 different distances (2, 4, and 6 cm) between the stimulating electrodes. While the cathode (1-cm wide and 5-cm long) was fixed to a point 6 cm proximal to the transverse crease of the wrist, the anode (1-cm wide and 5-cm long) was positioned at a distance of either 2, 4 or 6 cm from the cathode. Electrical stimulation was performed at the left forehand for 30 min at a frequency of 10 Hz.

### Experiment 4: Effects of stimulation of the median nerve on reducing BP

Small rectangular surface electrodes (size: 1.5-cm wide and 1.5-cm long) were placed over either the median nerve between the ligaments of the flexor carpi radialis and the palmaris longus (median site #1; interelectrode distance, 4 cm), collinear ulnar side (ulnar site; interelectrode distance, 4 cm) or palm side thumb (median site #2; interelectrode distance, 4 cm) to identify the nerve mediating the reduced BP. The stimulation was performed on the left forehand for 30 min at 10 Hz.

### Experiment 5: Microneurography to determine fiber types activated during TMNS

Microneurography was performed for the median nerve at the left forearm in 5 subjects (5 males) using previously described methods^36^ to determine which afferent fiber (A or C) was activated during TNMS. Briefly, a disposable tungsten electrode (Parylene, 0.005′, 1 M, A-M systems, WA, USA) was manually inserted through the skin into the median nerve 5 cm proximal to the distal crease of the left wrist. A reference electrode was attached 1–2 cm away. The neural signals were amplified 10000 times with a bioamplifier (WPI ISO-80, Sarasota, Florida, USA) and filtered between 300 and 10 kHz. Single-unit afferent activity was then isolated with a Micro1401 unit (CED, Cambridge, UK) and the Spike2 program (version 7.9; CED, Cambridge, UK). Action potentials were evoked by mechanically stimulating the skin of median nerve territory using a camel-hair brush, von Frey filament (8 g of force), wooden stick, or blunt-tipped forceps to ensure that the unit activity was derived from afferent fibers of the median nerve. The conduction velocity of the fibers was measured by dividing the conduction distances by the latency after the electrical stimulation artifact. The fiber types were classified according to the conduction velocity: >4 m/sec for A-fibers and <2 m/sec for C-fibers^37^. The rates of A- and C-fiber action potentials generated during the 10-sec TMNS (10 Hz) were measured.

### Experiment 6: Effect of an A-fiber block (AFB) during TMNS on reducing BP

#### Experiment 6-1: Induction and monitoring of an AFB of the median nerve

An AFB of the median nerve was induced and monitored in six subjects using a previously described method^11^ with slight modifications. The left hand was placed on a cushion in a position that was able to be comfortably maintained for approximately 2 hrs. Compressive pressure of 1.5 kg was applied to the skin over the median nerve 7 cm proximal to the wrist. The development of the AFB was evaluated by measuring the tactile threshold using von Frey filaments (Precise Tactile Sensory Evaluator, Anesthesio®, USA) applied in an up-down testing paradigm^38^ or the perception of warm sensations (number of warm sensations in response to twenty applications of hot water; 57 °C) to the palmar aspect of the thumb, index or middle fingers at 20-min intervals.

#### Experiment 6-2: Effect of an AFB on reducing elevated BP during TMNS

An AFB of the median nerve in the left hand was maintained for 80 min prior to TMNS in hypertensive subjects (n = 5) to determine whether median nerve A-fibers mediate the effects of TMNS. TMNS was applied to the left forehand for 30 min at a frequency of 10 Hz, and BP and HR were monitored for 120 min.

### Experiment 7: Effect of a topical capsaicin treatment on BP and HR in hypertensive subjects

Capsaicin (0.1% in ethanol; Sigma) was applied to an area of skin approximately 1.5 cm in diameter over the median nerve 6 cm proximal to the crease of the left wrist in hypertensive subjects (n = 5). Starting immediately after the application of topical capsaicin, the BP and HR were measured for 120 min.

### Experiment 8: Construction of a prototype device consisting of a TMNS unit and a wrist BP monitor

For the construction of a prototype device, a TMNS unit with optimal parameters for lowering BP was designed and combined with an automatic wrist BP monitor (BP629, Omron Healthcare Inc., Lake Forest, IL, USA).

### Data analysis

All data are presented as the mean ± SEM (standard error of mean) and were analyzed by one- or two-way repeated measurement analysis of variance (ANOVA) followed by Tukey’s post hoc tests unless indicated otherwise. Statistical significance was considered at p < 0.05.

## Electronic supplementary material

Supplementary figure 1-2
