# Supplementary material for: Attenuation of hypertension by C-fiber stimulation of the human median nerve and the concept-based novel device
Source: Sci Rep. 2018 Oct 8;8:14967. doi: 10.1038/s41598-018-33402-1 (PMC6175881; doi:10.1038/s41598-018-33402-1)
Supplement: Supplementary file 1 — Supplementary figure 1-2 [file 41598_2018_33402_MOESM1_ESM.pdf]

<Supplementary information>

Attenuation of hypertension by C-fiber stimulation of the human median nerve and the concept-based novel device

Authors: Se Kyun Bang, Yeonhee Ryu, Suchan Chang, Chae Kwang Im, Jong Han Bae, Young Seob Gwak, Chae Ha Yang, and Hee Young Kim

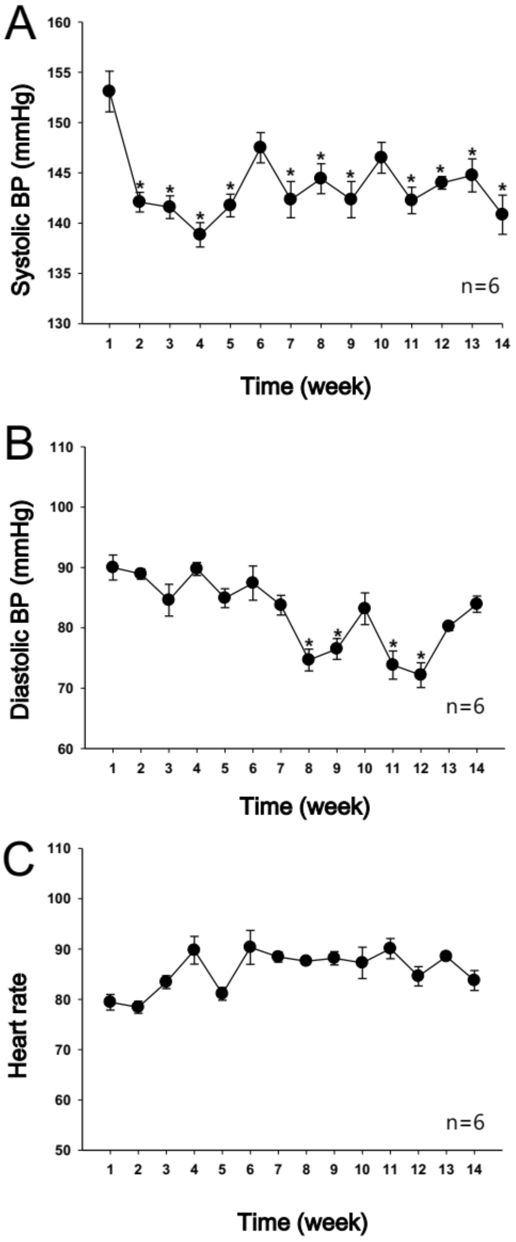

Supplementary Figure 1. Effects of repetitive TMNS on hypertension

Changes in basal systolic BP (A), diastolic BP (B) and HR (C) in the 6 participants who received repetitive TMNS over 14 weeks. *$P<0.05$ vs. the basal level before treatment (week 1).

CONSORT
TRANSPARENT REPORTING of TRIALS

CONSORT 2010 Flow Diagram

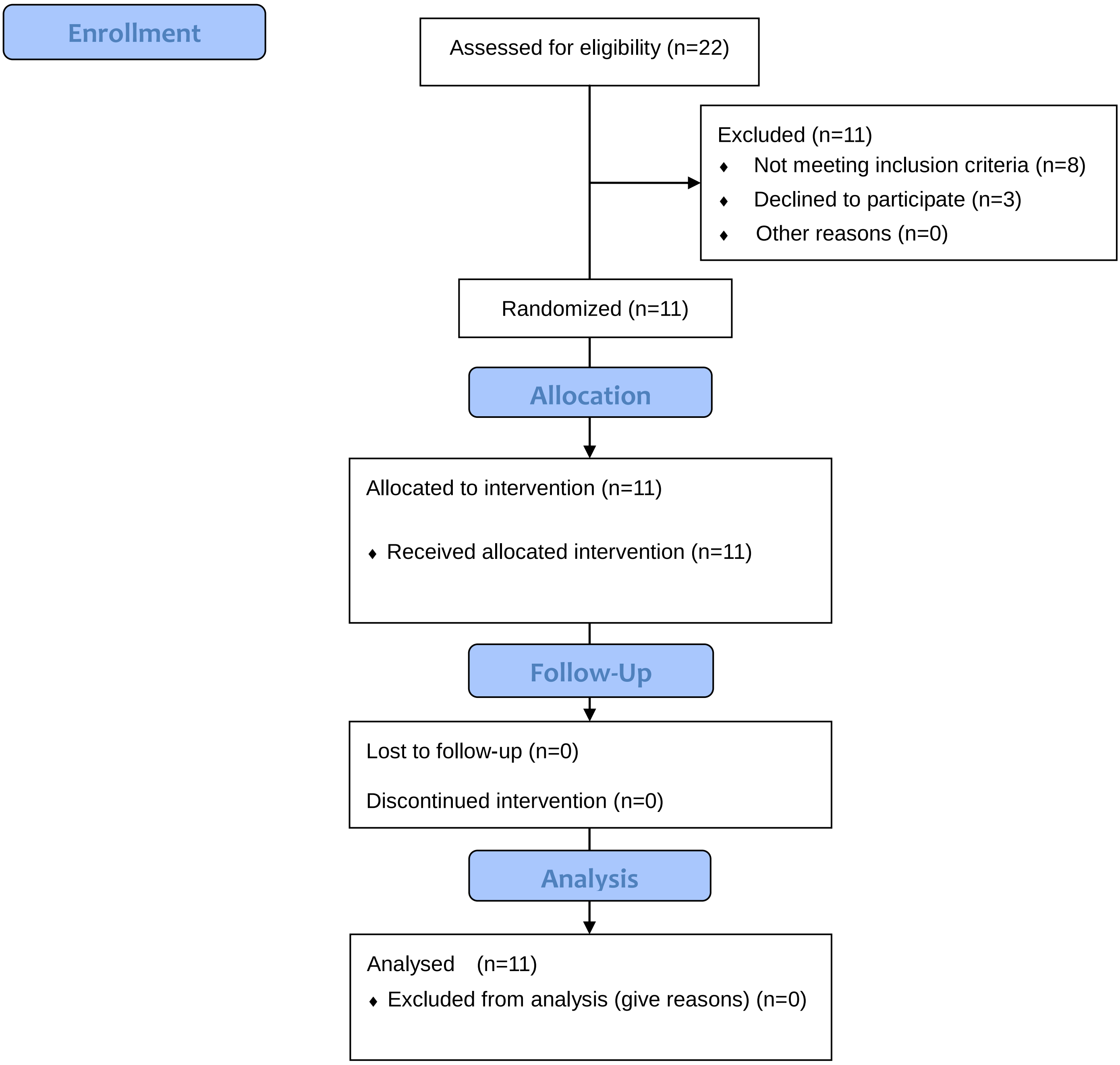

Supplementary figure 2. CONSORT 2010 Flow Diagram
